# Representation and participation across 20 years of plant genome sequencing

**DOI:** 10.1038/s41477-021-01031-8

**Published:** 2021-11-29

**Authors:** Rose A. Marks, Scott Hotaling, Paul B. Frandsen, Robert VanBuren

**Affiliations:** 1 Department of Horticulture, Michigan State University, East Lansing, MI USA; 2 Plant Resilience Institute, Michigan State University, East Lansing, MI USA; 3 Department of Molecular and Cell Biology, University of Cape Town, Rondebosch, South Africa; 4 School of Biological Sciences, Washington State University, Pullman, WA USA; 5 Department of Plant and Wildlife Sciences, Brigham Young University, Provo, UT USA; 6 Data Science Lab, Smithsonian Institution, Washington, DC USA

**Keywords:** DNA sequencing, Taxonomy, Plant genetics

## Abstract

The field of plant genome sequencing has grown rapidly in the past 20 years, leading to increases in the quantity and quality of publicly available genomic resources. The growing wealth of genomic data from an increasingly diverse set of taxa provides unprecedented potential to better understand the genome biology and evolution of land plants. Here we provide a contemporary view of land plant genomics, including analyses on assembly quality, taxonomic distribution of sequenced species and national participation. We show that assembly quality has increased dramatically in recent years, that substantial taxonomic gaps exist and that the field has been dominated by affluent nations in the Global North and China, despite a wide geographic distribution of study species. We identify numerous disconnects between the native range of focal species and the national affiliation of the researchers studying them, which we argue are rooted in colonialism—both past and present. Luckily, falling sequencing costs, widening availability of analytical tools and an increasingly connected scientific community provide key opportunities to improve existing assemblies, fill sampling gaps and empower a more global plant genomics community.

## Abstract (web summary)

Analyses of plant genomes sequenced in the past 20 years, the species taxonomic distribution and national participation reveal that genome quality has increased but substantial taxonomic gaps exist, and that the field has been dominated by the Global North.

## Main

The pace of sequencing and quality of land plant (Embryophyta) genome assemblies have increased dramatically over the past 20 years. Since the genome assembly of *Arabidopsis thaliana—*the first for any land plant—was published in 2000^1^, hundreds of plant genomes have been sequenced, assembled and made publicly available on GenBank^2^ and other repositories for genomic data. With large, complex genomes and varying levels of ploidy, plant genomes have been historically difficult to assemble. However, technological advances, such as long-read sequencing and new computational tools, have made sequencing and assembly of virtually any species possible^3–5^. Here, we provide an overview of the first 20 years of plant genome sequencing, including assessments of assembly quality, taxonomic representation and geographic participation.

Land plants are extremely diverse, with publicly available genome assemblies now spanning over ~500 million years of evolution^6–8^. However, only a small fraction (~0.16%) of the ~350,000 extant land plants have had their genome sequenced, and these efforts have not been evenly distributed across clades^9^. For some plants (for example, maize, *Arabidopsis* and rice^10–12^), multiple, high-quality genome assemblies are available and thousands of accessions, cultivars and ecotypes have been resequenced using high-coverage Illumina data^13^. Brassicaceae, a medium-sized plant family (~3,700 species^14^), is the most heavily sequenced, with genome assemblies for dozens of species including *Arabidopsis* and numerous cruciferous vegetables. In contrast, for most other groups, none or only a single species has a genome assembly. Ambitious efforts to fill taxonomic sampling gaps exist, including the Earth BioGenome and 10KP projects^15,16^, but individual research groups also play a role in expanding taxonomic representation in plant genomics.

With rapidly expanding resources and a new generation of scientists being trained, now is an ideal time to assess progress in terms of both taxonomic diversity and geographic representation in plant genome science. Economic disparities between nations, many of which were established due to colonialism, have a substantial impact on participation in science. Imperial colonialism provided scientists from the Global North access to a wealth of biodiversity, raw materials and ideas that would have been inaccessible to them otherwise^17–19^. Colonial scientists capitalized on this opportunity and, over time, this led to a disproportionate accumulation of wealth and scientific resources in the Global North^20^, which has contributed to the establishment and maintenance of global inequality^17,19,21^. Today, differences in funding, training opportunities, publication styles and language requirements continue to drive similar inequities^19,22–24^. In genomics, the high costs of sequencing and computational resources are barriers to entry that perpetuate existing imbalances established due to colonialism and economic disparities. Luckily, the diminishing cost and increasing accessibility of sequencing and computation infrastructure provide an opportunity to broaden participation and increase equity in genomics. This will require affluent nations and individuals to recognize their disproportionate access to biological and genetic resources and seek to increase participation rather than capitalizing on their privilege.

Here, we provide a high-level perspective on the first 20 years of genome sequencing in land plants. We describe the taxonomic distribution of sequencing efforts and build on previous estimates of genome availability and quality^25–28^. We show that an impressive and growing number of land plant genome assemblies are now publicly available, that quality has greatly improved in concert with the rise of long-read sequencing but that substantial taxonomic gaps exist. We also describe the geographic landscape of plant genomics, with an emphasis on representation. We highlight the need for the field, including its many affluent researchers and institutions, to work towards broadening participation. In our view, the wealth of publicly available genome assemblies can be leveraged to better understand plant biology while also continuing to decolonize a major field of research.

## Results

As of January 2021, 798 land plant species have genome assemblies. Six hundred and thirty-one of these were deposited in GenBank, and we identified a further 167 with genome assemblies via literature searches and cross-referencing against additional databases. If multiple genome assemblies were available for a species, we selected the highest-quality genome assembly (based on contiguity) as a representative for that species. Unless otherwise noted, all analyses were conducted on this dataset of 798 genome assemblies (Supplementary Table 1).

**Fig. 1:**
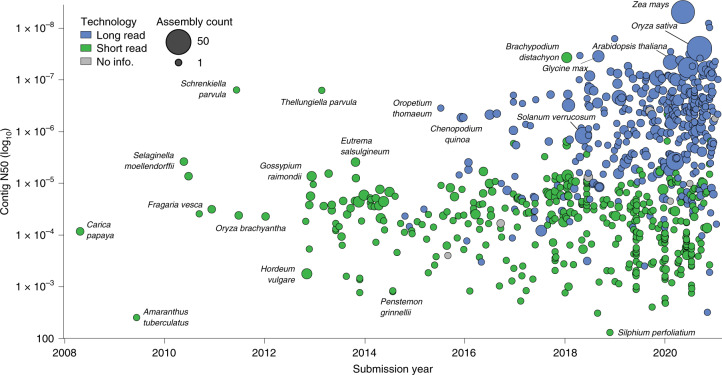
Changes in land plant genome assembly quality and availability over time. Assembly contiguity by submission date for 798 land plant species with publicly available genome assemblies. Points are coloured by the type of sequencing technology used and scaled by the number of assemblies available for that species. There is an improvement in contiguity associated with the advent of long-read sequencing technology, and a noticeable increase in the number of genome assemblies generated annually. All assemblies generated before 2008 have since been updated and are therefore not included.

The quantity and quality of land plant genome assemblies have increased rapidly, with particularly notable improvements associated with the advent of long-read sequencing (Fig. 1). Overall, 74% of land plant genome assemblies were produced in the past 3 years. Contig N50 (the length of the shortest contig in the set of contigs containing at least 50% of the assembly length) has also increased markedly in recent years, from 99.5 ± 48.1 kb in 2010 to 3,395.2 ± 735.4 kb in 2020. This increase appears to be driven primarily by advances in sequencing technologies. Assemblies constructed with short-read technology (for example, Illumina and Sanger) have significantly lower (*P* < 0.0001) contig N50 (124.6 ± 58.2 kb) compared to those that incorporate long reads (for example, PacBio and Oxford Nanopore) with a contig N50 of 4,033.4 ± 618.9 kb. This difference translates to an impressive ~32-fold increase in mean contig N50 for long-read assemblies. Nevertheless, many extremely fragmented plant genome assemblies have been published. Twenty-three of the assemblies in our dataset have a contig N50 <1 kb, and 158 with <10 kb.

**Fig. 2:**
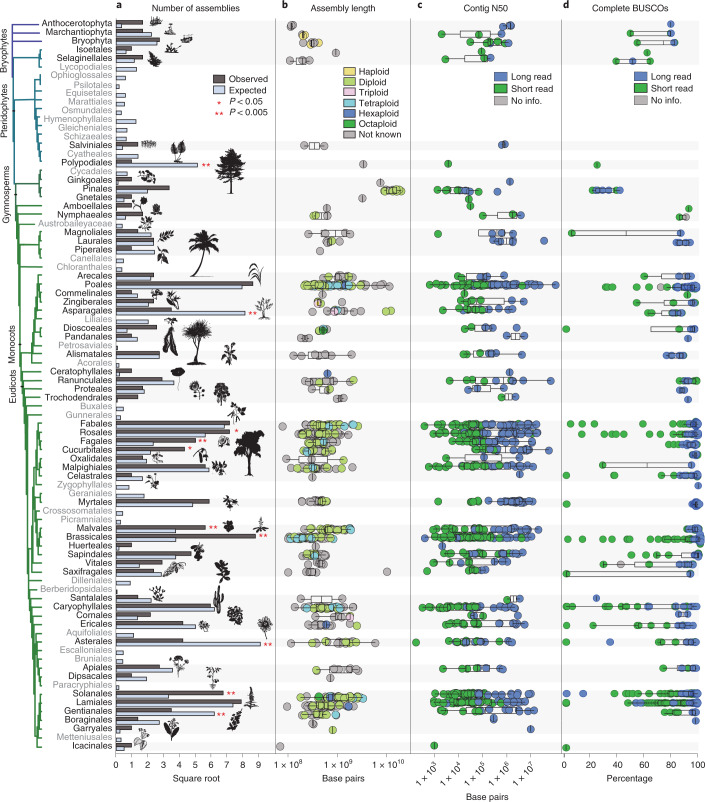
Comparison of genome availability and quality metrics for each land plant order. **a**, The number of species with publicly available genome assemblies as of January 2021 (*n* = 798) versus the number expected for each order. Significance values were calculated using Fisher’s exact test. Orders with no genome assemblies are shown in grey. Bryophytes are plotted at the phylum level, but Extended Data Fig. 2 shows bryophyte orders. Orders showing significant over- or under-representation are marked with asterisks. Over-represented orders include Brassicales (*P* = 3.03 × 10^–13^), Cucurbitales (*P* = 0.0038), Fagales (*P* = 0.0003), Malvales (*P* = 0.0084), Rosales (*P* = 0.0286) and Solanales (*P* = 1.27 × 10^–6^). Under-represented orders include Asparagales (*P* = 2.62 × 10^–11^), Asterales (*P* = 1.00 × 10^–10^), Gentianales (*P* = 0001) and Polypodiales (*P* = 8.93 × 10^–8^). **b**, Box plots showing the distribution of assembly length for each order of land plants. Points are coloured by ploidy. **c**, Box plots showing the distribution of contig N50 for each order of land plants. **d**, Box plots showing the distribution of complete BUSCO percentages for each order of land plants. **c**,**d**, Points are coloured by sequencing technology. For all box plots, the box defines the interquartile range (25th–75th percentile) and the centre line represents the median; whiskers extend to the maximum and minimum data values.

**Extended Data Fig. 1:**
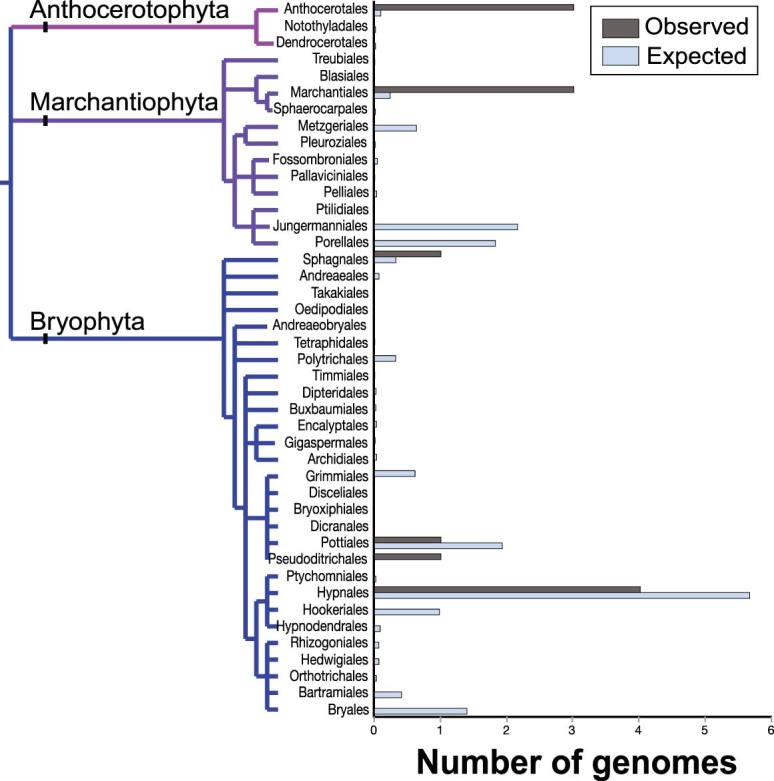
Statistical representation of bryophyte genome assemblies. The number of species in each bryophyte order with publicly available genome assemblies versus the number expected based on species richness. Significance values were calculated using Fishers Exact Tests. Orders without a genome assembly are shown in grey. Orders that showed a significant over- or under-representation are marked with ** (*P*  < 0.005) or * (*P* < 0.05).

**Extended Data Fig. 2:**
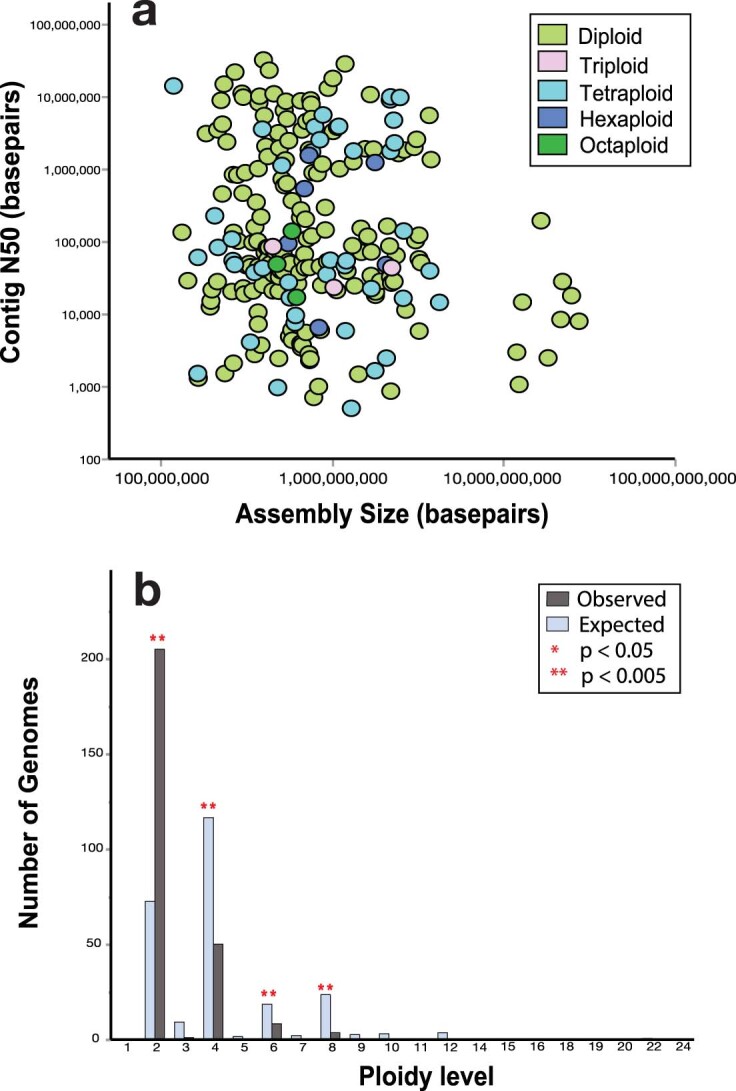
Quality and representation of polyploid assemblies. **a**, Genome assembly contiguity (N50) by assembly size for the 268 species with ploidy infromation. Contiguity is not associated with differences in genome size. The ploidy level of each genome is indicated by color. The mean N50s of polyploid and diploid genomes do not differ significantly. **b**, The observed vs. expected number of genome assemblies available for each ploidy level. Significance values were calculated using Fishers Exact Tests. Diploid genomes are statistically over-represented (*P* = 7.10e-11) and tetraploid (*P* = 3.13e-29), hexaploidy (*P* = 0.0465), and octoploid (*P* = 1.20e-04) genomes are statistically underrepresented. Ploidy levels that showed a significant over- or under-representation are marked with ** (*P* < 0.005) or * (*P* < 0.05).

The first land plants to have their genomes sequenced and assembled were model or crop species with small diploid genomes, but it is now feasible to assemble a genome for virtually any taxon. Nevertheless, taxonomic sampling gaps persist. Of the 137 land plant orders that have been described^29^, over half (76) lack a representative genome assembly. For the 62 orders with at least one genome assembly, a wide range of sampling depth is evident. For example, there are 83 species with genome assemblies in Brassicales, 80 in Poales and 67 in Lamiales, yet there are 41 orders with ten or fewer sequenced species. Six orders of land plants are statistically over-represented in genome assembly databases based on species richness. These include the agriculturally and economically important clades of Brassicales, Cucurbitales, Fagales, Malvales, Rosales and Solanales. Four orders of land plants had significantly fewer genome assemblies than expected based on species richness (Fig. 2). Not surprisingly, these were speciose orders with notable ecological but comparatively less economic importance—Asparagales, Asterales, Gentianales and Polypodiales (Fig. 2a). Bryophytes are poorly represented, with assemblies for only eight mosses, three liverworts and three hornworts (Fig. 2a and Extended Data Fig. 1). Diploid species are also statistically over-represented in terms of genome assembly availability (Fig. 2b and Extended Data Fig. 2) despite the widespread occurrence of polyploid plants^30^. Until recently, technological limitations have made it difficult to assemble high-quality polyploid genomes^4^. However, with the improvements offered by long-read sequencing, it is becoming more feasible to sequence and assemble large-polyploid plant genomes. As a result, there are some highly contiguous tetraploid and reasonably contiguous hexaploid genome assemblies, with mean contig N50 of 1,855.7 ± 474.3 and 251.9 ± 99.8 kb, respectively (Extended Data Fig. 2).

**Extended Data Fig. 3:**
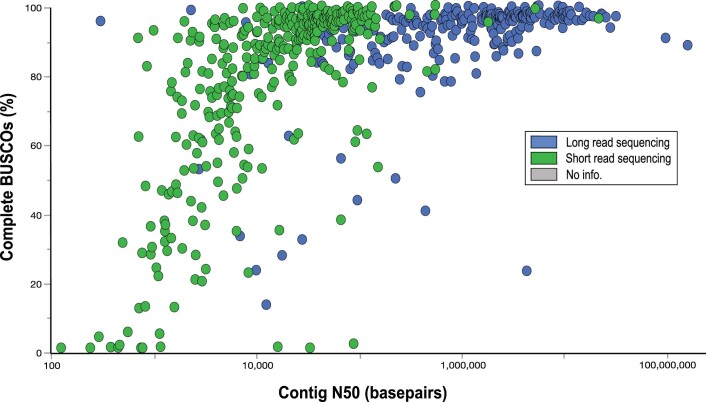
Relationship between assembly contiguity and the percentage of complete BUSCOs. Genome assembly contiguity is positively associated with the percent of complete BUSCOs identified (*n* = 627). Overall, assemblies generated with long-read sequencing capture a higher percentage of complete BUSCOs.

To further assess differences in assembly quality and completeness, we quantified the percentage of benchmarking universal single-copy orthologues (BUSCO, v.4.1.421) using the Embryophyta gene set from OrthoDB v.10 (ref. ^31^) that were present in each land plant genome assembly deposited in GenBank. There was a high degree of variability in BUSCO completeness: percentages of complete BUSCOs (single and duplicated genes) ranged from 0% to 99% across the available genome assemblies (Fig. 2d). More contiguous genome assemblies with higher contig N50 had more complete BUSCOs (*P* < 0.0088), and this was associated with the use of long reads in the assembly process (*P* < 0.0001; Fig. 2c–d and Extended Data Fig. 3). Despite the wide range of BUSCO completeness, no significant associations between the percentage of BUSCOs and genome size, taxonomy or domestication status were identified.

**Fig. 3:**
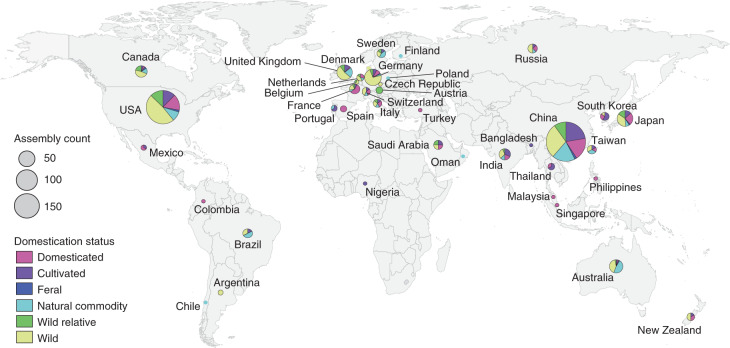
Geographic distribution of the submitting institutions for 798 plant genome assemblies. Circles are scaled by the number of genome assemblies produced in each nation and coloured by the relative proportion of domesticated, cultivated, feral, natural commodity, wild and wild relative species sequenced.

To quantify whether a bias exists towards sequencing economically important plants relative to other species, we classified the domestication status of each species with a genome assembly into six categories: (1) domesticated: plants that have undergone extensive artificial selection; (2) cultivated: plants that are used by humans but have not been subjected to substantial artificial selection; (3) natural commodity: plants that are harvested with little cultivation; (4) feral: plants that are not economically important but have still been influenced by human selection; (5) wild: plants that occur in the wild and have not been directly impacted by humans; and (6) wild relatives: wild plants that are closely related to or progenitors of domesticated and cultivated crops. Based on these categories, genome assemblies are available for 135 domesticated, 127 cultivated, 120 natural commodity and 12 feral species. The remaining 404 genome assemblies are from wild species; of these, 77 are wild relatives of crops (Fig. 3). While the number of human-linked species (that is, domesticated, cultivated, natural commodity and feral) with genome assemblies is largely equivalent to wild species, this equivalence reflects an extreme bias. There are far more wild (~350,000)^32^ than domesticated species (~1,200–2,000)^33,34^, suggesting that wild plants represent an untapped reservoir of genomic information.

To better understand global participation in plant genomics, we identified the submitting institution for each genome assembly in our dataset. If the submitting institution was not listed, we identified the corresponding author for the associated publication and assigned the genome to the location of that institution. While this approach does not account for secondary affiliations in other nations, it does reveal where most of the scientific credit for a genome assembly is probably placed. We find that land plant genome sequencing is dominated by China (235 assemblies), the USA (212 assemblies) and European nations (168 assemblies), with ~77% of genome assemblies attributed to one of those three regions (Fig. 3). Far fewer plant genome assemblies have been led by teams in Oceania (40 assemblies), South America (nine assemblies) and Africa (one assembly). These patterns probably reflect well-documented differences in training incentives, facilities and funding opportunities among nations^23,35–37^, many of which have been established and perpetuated through colonial practices^19^.

**Fig. 4:**
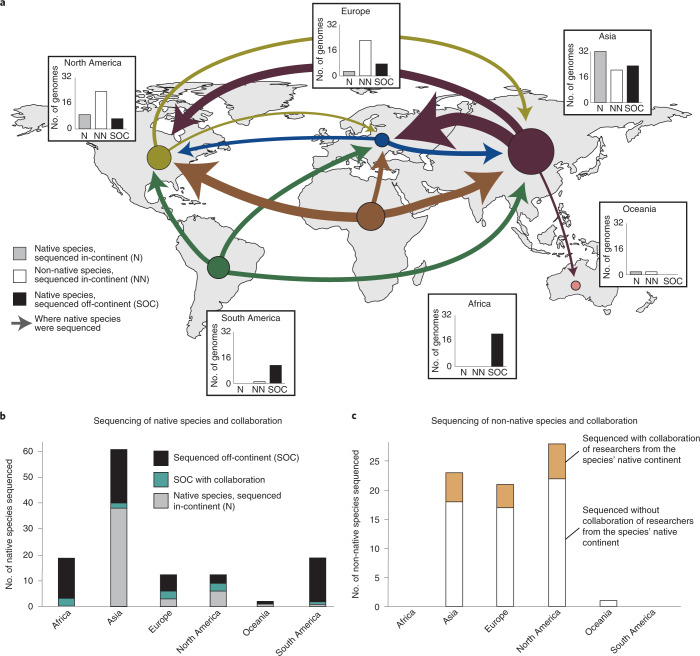
Disparities between species origin and lead sequencing institutions. **a**, Geographic perspective on where domesticated plants (*n* = 135) are native to versus where their genome assemblies were generated. Circle size and arrow weights are scaled by the number of genome assemblies represented. Circles represent the species native to that continent while arrows terminate in the continent where the species were sequenced. **b**, Number of domesticated species native to each continent and affiliations of the sequencing teams. **c**, Number of non-native species sequenced in each continent and the proportion of those efforts that included co-authors from the native range of the focal species.

Notably, many plant genome assemblies are for species that are native to, or have economic importance in, Africa and South America but have been sequenced by researchers elsewhere. We compared the centre of diversity^38^ for all 135 domesticated crops in our dataset with the location of the institution that led its genome sequencing. We also investigated the affiliations of co-authors to gain insight into the extent of international collaboration. Although we did not account for geographical patterns of contemporary cultivation, the findings shed light on a disconnect between the origin of many crops and the institutions leading their genomic research. We find that while there has been some reciprocal exchange between China, Europe and North America, nearly all crops native to Africa and South America have been sequenced off-continent; this represents a substantial global imbalance in genomics. There are dozens of major crops native to Africa and South America represented in GenBank, yet only one (*Phaseolus lunatus*) has a primary affiliation in South America and none were led by African institutions (Fig. 4). Even when co-author affiliations and collaborations are taken into account, this pattern holds true: most crops native to Africa and South America have been sequenced off-continent by non-collaborative teams. In general, plant genome sequencing projects are led and conducted exclusively in China, Europe and the USA.

## Discussion

The field of plant genomics has grown rapidly in the past 20 years, giving rise to an array of new tools, datasets and biological insights. The quality of genome assemblies being produced today is much improved compared to even a few years ago, and this trend shows no signs of slowing. As has been observed for insects^39^, the improvement in plant genome assembly quality appears to be driven largely by increased use of long-read sequences in assemblies. These technologies have enabled assembly of increasingly complex and polyploid genomes, opening up new arenas of research for plant genome scientists. Despite these advances, major biases exist in both taxonomic representation and geographic participation. As the field continues to grow, there is an opportunity to fill key taxonomic gaps and build a broader, more representative discipline.

To date, plant genome scientists have emphasized sequencing of economically important and model species with small diploid genomes. This has led to major agricultural breakthroughs and fundamental scientific insights, and these densely sampled clades are ideal systems for investigation of intraspecific variation and pan-genome structure. However, this approach has overlooked the wealth of information contained within the genomes of wild plants, which are extremely diverse and largely untapped. Wild plants exhibit numerous diverse properties and produce a wide range of secondary compounds, many of which have important traditional and emerging pharmaceutical and industrial applications^40^. Numerous medical therapeutics and commercial materials are derived from, or made to mimic, plant-based compounds^41^ yet we have only begun to explore the rich chemical diversity of wild plants. Given the rapid loss of global biodiversity, it is critical that we take the opportunity to learn what we can from wild species before they disappear. Over the past ~100 years we have witnessed a 60% increase in plant extinction^42^ and, despite conservation efforts, this loss of biodiversity is projected to continue even under the most optimistic scenarios^43^. We urge researchers to take advantage of new genomic technologies that provide an opportunity to explore, catalogue and mine the immense diversity of information contained within wild species before they are lost.

In addition to taxonomic gaps, participation gaps are also prevalent in land plant genomics. The field is dominated by a handful of affluent nations primarily from the Global North (for example, the USA, Germany and the UK) and China. In addition, our analyses reveal a discrepancy between the native ranges of species and where their genomes have been sequenced and assembled. In fact, 56% of all domesticated crops have had their genome sequenced outside of their continent of origin and only 13% of these included in-continent collaborators (Fig. 4). Much of the evolutionary innovation observed in landraces, locally adapted cultivars and wild plants is exclusively maintained in the Global South, but only a handful of genome assemblies have been led by groups in those regions (except for China, a notable economic and technological outlier relative to other nations of the Global South; Fig. 4). The lack of international collaboration is concerning since, in some instances of off-continent genomics, it is likely that the sequenced material was chosen with minimal input from local stakeholders. Thus, the resulting genome assemblies may not represent the germplasm grown in production regions and the analyses may not address grower priorities. That being said, there are a growing number of inclusive and collaborative plant genomics projects such as the Orphan Crop Genome Consortium (http://africanorphancrops.org) and Africa BioGenome Project that are building capacity and broadening participation in genomics^23^.

We argue that these dynamics are rooted in historical colonialism and economic barriers to entry and are being perpetuated by contemporary ‘parachute science’. Historically, science was intimately linked to the rise of imperial colonialism^17–19^. Innovations in navigation and cartography enabled conquest of new territories by nations in the Global North, and scientific curiosity actually motivated many early colonial expeditions^17^. Once colonies were established, they became the first sites for parachute science. Imperial scientists would travel to colonies, make collections and take credit for their ‘discoveries’, often discounting indigenous knowledge in the process. Over time, this led to a disproportionate accumulation of wealth, both scientific and economic, in the Global North that continues to drive disparities and participation imbalances in science today^19–21^. While historical colonialism set the stage for European nations to consolidate wealth and biological resources, both China and the USA have colonized surrounding territories in modern times. The resulting economic privilege has allowed these nations to capitalize on biological and genomic resources globally. Despite outward criticism of colonialism and legal provisions aimed at preventing international transport of biological and genetic resources (for example, the Nagoya Protocol), affluent nations continue to lead bio- and genomic-prospecting efforts and parachute science remains prevalent^44,45^.

Going forward, we recommend that local communities and indigenous knowledge associated with the global reservoir of plant diversity^46,47^ form the backbone of plant genome collaborations. Currently there are over a dozen plant genomics projects with African institutions as partners^23^, a growing number of projects integrating indigenous knowledge^46,48^, large-scale consortia with multinational participants are being established (for example, the Africa BioGenome Project) and some journals have implemented policies to minimize parachute science and encourage international collaboration (for example, PLOS’s policy on inclusion in global research). These efforts all stand to broaden participation in plant genomics. As North American scientists, we acknowledge our own implicit—sometimes explicit—participation in the sequencing and analysis of non-native plants. We encourage all plant scientists to strive to support local stakeholders, to incorporate indigenous knowledge into their work and to invest in building systems and expertise for working with genomic resources in the location where they occur naturally. We believe that in-continent institutions should be encouraged to lead genomic research of native species.

Plant genome science has arrived at an exciting moment, with a rapidly expanding pool of genomic resources being generated by an increasingly diverse group of scientists. However, to take full advantage of the opportunities that a modern discipline affords and to ensure that the field continues striving for equity, we offer three recommendations. (1) Plant genome scientists should embrace long-read sequencing technologies and leverage them whenever possible to generate new assemblies. This is already occurring but, given the massive disparity in quality between assemblies generated with short-read versus long-read data, the need for continued adoption cannot be overstated. (2) Despite considerable progress, the taxonomic scope and domestication status of plants with available genome assemblies should continue to be expanded. In our view, attention should be focused on generating assemblies for clades that have none (for example, Hymenophyllales, Cyatheales, Geraniales and Dilleniales; Fig. 2a), adding more complex plant genome assemblies (for example, large, repetitive and/or polyploid) and sequencing wild species. (3) While the progress driven by large-scale consortia is undeniable, it is important that researchers in the discipline are mindful of the signatures of colonialism—both past and present—in plant genome science. To this end, we should collectively monitor consortia, collaborations and projects to ensure that ethical approaches are being taken, in-country peoples are given a voice and that participation and access to resources is broadened at every level. Ultimately, a diverse, thriving discipline with empowered researchers across continents, regardless of socioeconomic status, will yield the greatest potential to meet the economic, social and evolutionary challenges facing twenty-first-century plant science.

## Methods

A complete list of the species and associated metadata analysed in this study is provided in Supplementary Table 1. To compile a list of the optimal genome assemblies for all land plants, we first downloaded the most contiguous genome assembly for each species represented in GenBank in January 2021. Genome assemblies were downloaded using the *download-genome* function of NCBI’s datasets tool (v.10.9.0), and metadata were extracted using the *assembly-descriptors* function of NCBI’s datasets tool. Data on sequencing technology, coverage, assembler and submitting institution were retrieved using the python (v.3.7.9) script *scrape_assembly_info.py* (https://github.com/pbfrandsen/insect_genome_assemblies). For genome assemblies with no reported sequencing technology on GenBank, we went to the publication associated with the assembly (if available) and identified the sequencing technology from the reported methods. Subsequently, we conducted an extensive literature search to identify additional genome assemblies not deposited in GenBank. To do so, we took advantage of review papers summarizing plant genome assemblies^25–28^ and other datasets such as PlaBi database (https://www.plabipd.de), Phytozome (https://phytozome.jgi.doe.gov/), Fernbase (https://www.fernbase.org) and Wikipedia (https://en.wikipedia.org/wiki/List_of_sequenced_plant_genomes). We cross-referenced these datasets against NCBI to develop a non-redundant but comprehensive list of land plant genome assemblies. For genome assemblies not deposited in NCBI, metadata (including assembly size, contig N50, sequencing technology, authorship and domestication status) were manually extracted from the primary publication.

Higher-level taxonomy for each species was integrated with taxonkit (v.0.8.0)^49^. To place species in a phylogenetic context, we identified the most up-to-date phylogenies for each major group of land plants and grafted them together. For angiosperms we used the APG IV tree^50^, for gymnosperms and pteridophytes we used the APGweb tree (http://www.mobot.org/MOBOT/research/APweb) and for bryophytes we used iTol (v.4)^51^. Many of the relationships among these groups are still poorly resolved or under ongoing revision but, for the purposes of this work, they are sufficient to visualize general relationships among clades.

To identify cases where the observed number of genome assemblies for an order differed significantly from the number expected based on species richness, we tested for over- or under-representation of genome assemblies in each land plant order using Fisher’s exact test in R (v.4.1.0). To do so, we compiled a list of the total numbers of species in each land plant order. For vascular plants, we used the Leipzig Catalogue of Vascular Plants (v1.0.3)^29^ in combination with the summaries provided in ref. ^52^. For bryophytes, we compiled data from the Plant List (http://www.theplantlist.org; accepted names only) and cross-referenced these against the Missouri Botanical Gardens Index of Bryophytes (http://www.mobot.org/mobot/tropicos/most/bryolist.shtml). Next, we computed the number of genome assemblies that would be expected for each order if sampling effort was evenly distributed. We then ran Fisher’s exact test in R (v.4.1.0) to identify clades with statistical over- or under-representation of genome assemblies.

To quantify the distribution of polyploid genome assemblies, we pulled data on chromosome number and ploidy from the Kew Botanical Gardens Plant DNA C-values database^53^. In total, this database contained entries for 268 species with sequenced genomes. We did not collect metadata on ploidy for the remaining sequenced genomes because this is not always clear or readily available in the associated publications. These data were used to calculate the total number of species with each ploidy level. We then calculated the number of genome assemblies expected for every ploidy level and ran Fisher’s exact test in R (v.4.1.0) to identify over- or under-represented ploidy levels.

We classified the domestication status of each species in our dataset using a six-category scale. Each species was designated as either (1) domesticated: plants that have undergone extensive artificial selection; (2) cultivated: plants that are used by humans but have not been subjected to substantial artificial selection; (3) natural commodity: plants that are naturally harvested with little cultivation; (4) feral: plants that are not economically important but have still been influenced by human selection; (5) wild: plants that occur in the wild and have not been directly impacted by humans; and (6) wild relatives: plants that are closely related to domesticated or cultivated crops. Using this classification system, we computed the total number of genome assemblies for each category.

We investigated the completeness of each genome assembly by quantifying the percentage of complete, fragmented and missing BUSCOs (v.4.1.4) from the Embryophyta gene set in OrthoDB (v.10)^31^. We ran BUSCO (v.4.1.4) in *–genome mode* on each GenBank assembly with the *–long* option. We did not include genome assemblies gathered from published papers in these analyses due to difficulties in accessing the genome files. We tested for an association between the percentage of complete BUSCOs (single and duplicated) and the contiguity of genome assemblies (contig N50) using a linear model in R (v.4.1.0). Similarly, we tested for an effect of sequencing technology on the percentage of complete BUSCOs using a linear model in R (v.4.1.0), with assembly size included as a random effect.

To estimate the geographic distribution of plant genome projects, we identified the submitting institution for each genome assembly in our dataset. If the submitting institution was not listed, we identified the corresponding author for the publication and assigned the genome to the location of that institution. Next, we compiled data on the centre of diversity^38^ for all 135 domesticated crops with genome assemblies. For these species we dissected authorship in more detail, to account for collaborative efforts. We looked at the affiliations of all co-authors on each publication relative to the centre of diversity of the sequenced species. Projects were scored as either ‘in-continent team’, ‘off-continent team’, ‘led by off-continent team, with in-continent contributions’ or ‘led by in-continent team, with off-continent contributions’. Using these categories, we summarized global patterns of plant genome sequencing relative to the centre of origin for these important crops.

### Reporting Summary

Further information on research design is available in the Nature Research Reporting Summary linked to this article.

## Supplementary information

Reporting Summary

Supplementary Table 1 Table listing all genome assemblies and associated metadata analysed in the current study.

## Data Availability

All metadata associated with this project can be found in Supplementary Table 1. Accession numbers for all genome assemblies are also listed in Supplementary Table 1. Genome assemblies and associated publications can be accessed at GenBank (https://www.ncbi.nlm.nih.gov/genbank/), PlaBi database (https://www.plabipd.de/), Phytozome (https://phytozome.jgi.doe.gov/), Fernbase (https://www.fernbase.org/) and Wikipedia (https://en.wikipedia.org/wiki/List_of_sequenced_plant_genomes).

## Code availability

The primary code used in this study was modified for plants from https://github.com/pbfrandsen/insect_genome_assemblies.
