# Supplementary material for: Representation and participation across 20 years of plant genome sequencing
Source: Nat Plants. 2021 Nov 29;7(12):1571–8. doi: 10.1038/s41477-021-01031-8 (PMC8677620; doi:10.1038/s41477-021-01031-8)
Supplement: Supplementary file 1 — Reporting Summary [file 41477_2021_1031_MOESM1_ESM.pdf]

nature portfolio

Corresponding author(s): Rose A. Marks

Last updated by author(s): Oct 21, 2021

Reporting Summary

Nature Portfolio wishes to improve the reproducibility of the work that we publish. This form provides structure for consistency and transparency in reporting. For further information on Nature Portfolio policies, see our Editorial Policies and the Editorial Policy Checklist.

Statistics

For all statistical analyses, confirm that the following items are present in the figure legend, table legend, main text, or Methods section.

n/a	Confirmed	
☐	☒	The exact sample size (*n*) for each experimental group/condition, given as a discrete number and unit of measurement
☒	☐	A statement on whether measurements were taken from distinct samples or whether the same sample was measured repeatedly
☐	☒	The statistical test(s) used AND whether they are one- or two-sided<br>*Only common tests should be described solely by name; describe more complex techniques in the Methods section.*
☐	☒	A description of all covariates tested
☒	☐	A description of any assumptions or corrections, such as tests of normality and adjustment for multiple comparisons
☐	☒	A full description of the statistical parameters including central tendency (e.g. means) or other basic estimates (e.g. regression coefficient) AND variation (e.g. standard deviation) or associated estimates of uncertainty (e.g. confidence intervals)
☐	☒	For null hypothesis testing, the test statistic (e.g. *F*, *t*, *r*) with confidence intervals, effect sizes, degrees of freedom and *P* value noted<br>*Give P values as exact values whenever suitable.*
☒	☐	For Bayesian analysis, information on the choice of priors and Markov chain Monte Carlo settings
☒	☐	For hierarchical and complex designs, identification of the appropriate level for tests and full reporting of outcomes
☒	☐	Estimates of effect sizes (e.g. Cohen's *d*, Pearson's *r*), indicating how they were calculated

Our web collection on statistics for biologists contains articles on many of the points above.

Software and code

Policy information about availability of computer code

Data collection	A complete list of the genome assemblies and associated metadata used in this study are provided in Table S1. Genome assemblies were downloaded from GenBank using the download-genome function of NCBI's datasets tool (v.10.9.0) and metadata were extracted using the assembly-descriptors function of NCBI's datasets tool. Data on sequencing technology, coverage, assembler, and submitting institution were retrieved using the python (v.3.7.9) script scrape_assembly_info.py (https://github.com/pbfrandsen/insect_genome_assemblies). Higher level taxonomy for each species was integrated with taxonkit (v.0.8.0). We summarized species number for each land plant order from the Leipzig Catalogue of Vascular Plants (LCVP; v1.0.3), the Plant List (http://www.theplantlist.org; accepted names only), and the Missouri Botanical Gardens Index of Bryophytes (http://www.mobot.org/mobot/tropicos/most/bryolist.shtml). Plant phylogenies were compiled used the APG IV tree, the APGweb tree (http://www.mobot.org/MOBOT/research/APweb), and iTol (v.4). We pulled data on chromosome number and ploidy from the Kew Botanical Garden's Plant DNA C-values database.
Data analysis	We computed the number of genome assemblies that would be expected for each order if sampling effort was evenly distributed and ran Fisher's Exact Tests in R (v.4.1.0) to identify clades with a statistical over- or under- representation of genome assemblies. We then calculated the number of genome assemblies expected for every ploidy level and ran Fisher's Exact Tests in R (v.4.1.0) to identify over- or under-represented ploidy levels. We ran BUSCO (v.4.1.4) with the Embryophyta gene set in --genome mode with the --long option specified to quantify the percentage of complete, fragmented, and missing BUSCOs in each GenBank assembly. We tested for an association between the percentage of complete BUSCOs (single and duplicated) and the contiguity of genome assemblies (contig N50) using a linear model in R (v.4.1.0). Similarly, we tested for an effect of sequencing technology on the percentage of complete BUSCOs using a linear model in R (v.4.1.0) with assembly size included as a random effect.

For manuscripts utilizing custom algorithms or software that are central to the research but not yet described in published literature, software must be made available to editors and reviewers. We strongly encourage code deposition in a community repository (e.g. GitHub). See the Nature Portfolio guidelines for submitting code & software for further information.

Data

Policy information about availability of data

All manuscripts must include a data availability statement. This statement should provide the following information, where applicable:

- Accession codes, unique identifiers, or web links for publicly available datasets
- A description of any restrictions on data availability
- For clinical datasets or third party data, please ensure that the statement adheres to our policy

All metadata associated with this project can be found in Table S1. Accession numbers for all genome assemblies are also listed in Table S1. Genome assemblies and associated publications can be accessed at GenBank (https://www.ncbi.nlm.nih.gov/genbank/) , PlaBi database (https://www.plabipd.de/), Phytozome (https://phytozome.jgi.doe.gov/), Fernbase (https://www.fernbase.org/), and Wikipedia (https://en.wikipedia.org/wiki/List_of_sequenced_plant_genomes).

Field-specific reporting

Please select the one below that is the best fit for your research. If you are not sure, read the appropriate sections before making your selection.

☒ Life sciences ☐ Behavioural & social sciences ☐ Ecological, evolutionary & environmental sciences

For a reference copy of the document with all sections, see nature.com/documents/nr-reporting-summary-flat.pdf

Life sciences study design

All studies must disclose on these points even when the disclosure is negative.

Sample size	The sample size was determined by the number of publicly available plant genome assemblies. All 798 species with a representative genome assembly were included.
Data exclusions	Duplicate genome assemblies for a single species were exuded.
Replication	The degree of replication depended on the number of genome assemblies available for each order of land plants, ploidy level, sequencing technology, and BUSCO score.
Randomization	Our study provides a summary of publicly available genome assemblies and does not include any de-novo experiments. Samples were assigned to groups for statistical analyses based on associated metadata such as their phylogenetic order, ploidy level, sequencing technology, and BUSCO score.
Blinding	Because we analyzed metadata from previously published studies, we did not include any blinding measures.

Reporting for specific materials, systems and methods

We require information from authors about some types of materials, experimental systems and methods used in many studies. Here, indicate whether each material, system or method listed is relevant to your study. If you are not sure if a list item applies to your research, read the appropriate section before selecting a response.

Materials & experimental systems

n/a	Involved in the study
☒	☐ Antibodies
☒	☐ Eukaryotic cell lines
☒	☐ Palaeontology and archaeology
☒	☐ Animals and other organisms
☒	☐ Human research participants
☒	☐ Clinical data
☒	☐ Dual use research of concern

Methods

n/a	Involved in the study
☒	☐ ChIP-seq
☒	☐ Flow cytometry
☒	☐ MRI-based neuroimaging
